# P-1509. *In Vitro* Activity of Aztreonam-Avibactam Against Enterobacterales Isolated from Pediatric and Adult Patients Collected During the ATLAS Global Surveillance Program, 2018-2022

**DOI:** 10.1093/ofid/ofae631.1678

**Published:** 2025-01-29

**Authors:** Mark Estabrook, Julie Dickson, Gregory Stone, Katherine Perez, Daniel F Sahm

**Affiliations:** IHMA, Schaumburg, Illinois; IHMA, Schaumburg, Illinois; Pfizer, Inc., Groton, Connecticut; Pfizer, Inc., Groton, Connecticut; IHMA, Schaumburg, Illinois

## Abstract

**Background:**

The spread of antimicrobial resistance among clinically isolated Enterobacterales (Eba) is a threat to public health. Aztreonam (ATM) is a monobactam stable against hydrolysis by metallo-β-lactamases (MBLs) and avibactam (AVI) inhibits class A, class C, and some class D serine β-lactamases. ATM-AVI is being developed for use against infections caused by drug-resistant Eba, especially those co-producing MBLs and other β-lactamases. This study evaluated the *in vitro* activity of ATM-AVI and comparators against Eba collected in 2018-2022 from pediatric and adult patients as part of the ATLAS global surveillance program.

**Figure:**
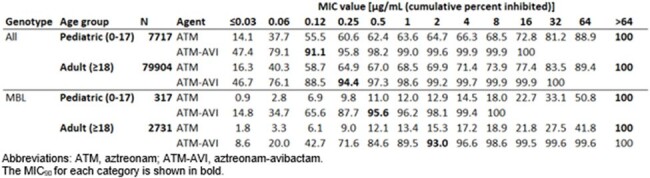

**Methods:**

87621 Eba isolates were collected from patients in 226 medical centers in 57 countries in Europe, Latin America, Asia/Pacific (excluding mainland China), and Middle East/Africa. Susceptibility testing was performed by CLSI broth microdilution and interpreted using CLSI 2024 breakpoints. PCR and sequencing were used to identify β-lactamase genes among all isolates testing with meropenem MIC >1 µg/mL, and a randomly sampled subset of approximately 80% of *Escherichia coli*, *Klebsiella* spp. and *Proteus mirabilis* testing with ATM or ceftazidime MIC >1 µg/mL.

**Results:**

MIC_90_ values for ATM-AVI of 0.12 µg/mL (pediatric isolates) and 0.25 µg/mL (adult isolates) were observed. Against all Eba isolates, ≤8 µg/mL of ATM-AVI inhibited 99.9% of both pediatric and adult isolates, whereas only 66.3% (pediatric) and 71.4% (adult) of these isolates were susceptible to ATM alone (table). Among isolates that screened positive for an MBL, MIC_90_ values for ATM-AVI were 0.5 µg/mL (pediatric) and 2 µg/mL (adult) and ATM-AVI inhibited 100% (pediatric) and 98.6% (adult) at concentrations ≤8 µg/ml. In contrast, only 14.5% (pediatric) and 17.2% (adult) of MBL-positive isolates were susceptible to ATM without AVI.

**Conclusion:**

Based on MIC_90_ values, ATM-AVI demonstrated potent *in vitro* activity against Eba isolated both from pediatric and adult patients. Avibactam’s ability to potentiate aztreonam against MBL-positive Enterobacterales isolates warrants its continued development.

**Disclosures:**

**Mark Estabrook, MS**, Pfizer, Inc.: Advisor/Consultant **Julie Dickson, BS**, Pfizer, Inc.: Advisor/Consultant **Daniel F. Sahm, PhD**, Pfizer, Inc.: Advisor/Consultant

